# Sleeve gastrectomy and one-year outcomes: Impact on cardiovascular, renal and metabolic parameters

**DOI:** 10.1016/j.sopen.2024.01.004

**Published:** 2024-01-12

**Authors:** Maria Irene Bellini, Lidia Castagneto Gissey, Denise V. Nemeth, Vito D'Andrea, Giulio Illuminati, Serena Marchitelli, Silvia Lai, Giovanni Casella

**Affiliations:** a Department of Surgery, Sapienza University of Rome, 00161 Rome, Italy; b School of Osteopathic Medicine, University of the Incarnate Word, San Antonio, TX 78235, USA; c Department of Experimental Medicine, Sapienza University of Rome, 00161 Rome, Italy; d Department of Translational and Precision Medicine, Sapienza University of Rome, Rome, Italy

**Keywords:** Bariatric surgery, Sleeve gastrectomy, Renal function, Cardiovascular disease

## Abstract

**Background:**

Cardiovascular and renal diseases represent a major determinant for the morbidity and mortality associated with obesity and type 2 diabetes mellitus (T2DM). Bariatric surgery is considered one of the few treatments with the potential to reverse cardiovascular, renal and metabolic disease.

**Methods:**

Prospective study of patients undergoing sleeve gastrectomy collecting pre- and post-surgery creatinine, eGFR, glucose, insulin, total, LDL/HDL cholesterol, triglycerides, parathyroid hormone, vitamin D3, C- Reactive Protein (CRP), blood count, weight, body mass index (BMI), bilateral carotid intima media thickness (IMT), flow-mediated dilation (FMD) and epicardial adipose tissue (EAT). Measurements were compared at 1 year follow up.

**Results:**

24 patients were included in the study. Cardiovascular parameters, as HDL-cholesterol (*p* = 0.002), IMT (*p* = 0.003), EAT (*p* < 0.001) and FMD (*p* = 0.001) showed significant improvement after surgery. Secondary renal outcomes including Vitamin D3 (*p* < 0.0001), Calcium (*p* = 0.006), RBCs (*p* = 0.007), HCO3- (*p* = 0.05) also ameliorated as well as BMI (*p* < 0.001).

**Conclusions:**

Sleeve gastrectomy has a positive impact on cardiovascular, renal, and metabolic parameters in patients with morbid obesity, suggesting it may halt the progression of these diseases even in the preclinical stage. Further research is needed to explore the long-term effects underlying these improvements.

## Introduction

Cardiovascular and renal diseases represent a major determinant for the morbidity and mortality associated with obesity and type 2 diabetes mellitus (T2DM) [1].

Bariatric surgery (BS) interferes with several aspects of the pathophysiology of cardiorenal syndrome in obesity [2] and is considered one of the few effective treatments to improve long-term cardiovascular and renal complications [3,4].

Structural, hemodynamic, and metabolic alterations for which obesity has been hypothesized as the major responsible [5], can cause severe damage to the kidney [6], namely glomerular hypertension, hypertrophy, and hyperfiltration, and eventually leading to progressive glomerulosclerosis and loss of function [7]. These renal complications are seen in obesity-related glomerulopathy and represent the expression of a more generalized systemic damage reflecting an underlying chronic inflammatory state [8], as well as a direct effect of adiposity [9], for which, in the high body mass index (BMI) population, cardiovascular risk significantly increased [28].

In view of the fact that cardiovascular and renal diseases account for a significant proportion of the morbidity and excess mortality associated with obesity and that the timeframe for BS to halt this progression is limited, the ideal intervention should take place before there is clinical evidence of the disease [10]. When in fact the above mentioned changes become clinically significant, the chances they could be reversible decrease by time, with for example obese end stage kidney disease patients being denied access to transplantation because of their weight, although in the meanwhile they face significant difficulties in embracing rehabilitation programs, because of the imposed limitations by dialysis [11].

**Fig. 1:**
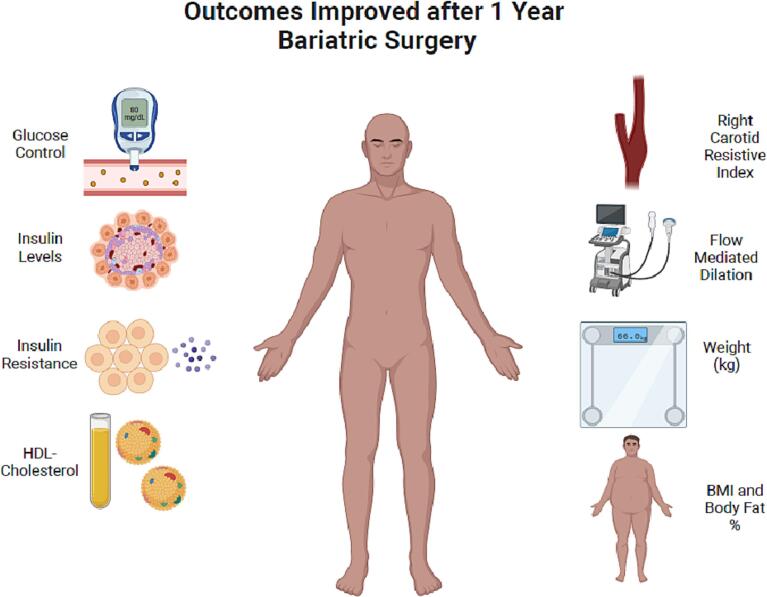
Cardiac, renal and metabolic parameters measured.

The aim of this study is to assess the impact of BS for the treatment of cardiovascular, renal and metabolic diseases in the context of obesity, to possibly highlight the pre-emptive damage reversal, by analysing how structural measures, metabolic parameters, and pre-specified cardiovascular and renal outcomes vary after surgery (Fig. 1).

## Patients and methods

### Study design

This is a prospective cohort study designed to evaluate the short-term effects of sleeve gastrectomy (SG) on cardiovascular, renal and metabolic outcomes. The experimental protocol was approved by the Ethical Committee of the Department of Surgery at Sapienza University. Informed consent was obtained from all participants.

A total of 24 patients affected by morbid obesity and consecutively undergoing SG at our institution were included in the study. The selection of these subjects was not randomised. A clinical assessment was proposed to all patients, who were informed about the follow-up benefits, in terms of prevention and early treatment of any detected condition. Participants were followed-up for 12 months to assess the short-term effects of SG on analysed outcomes. Patient inclusion criteria comprised those enlisted by international guidelines [12]. Subjects who underwent revision bariatric surgery or who had end stage renal disease or severe cardiovascular conditions were excluded from the study.

All patients underwent a standard preoperative multidisciplinary workup following institutional, national and international guidelines, involving complete history and physical examination, routine laboratory tests, chest X-ray, abdominal ultrasonography, esophagogastroduodenoscopy and nutritional and psychiatric assessment. Furthermore, preoperative and postoperative values for the following laboratory tests were recorded: Creatinine, eGFR, BUN, glucose, insulin, Homeostatic Model Assessment for Insulin Resistance (HOMA-IR), Total Cholesterol, LDL Cholesterol, HDL Cholesterol, Triglycerides, Parathyroid hormone, Vitamin D3 levels, C-Reactive Protein (CRP), haemoglobin, Mean Corpuscular Volume (MCV), mean corpuscular haemoglobin concentration (MCHC), hematocrit (Hct), transferrin, red blood count, white blood count, platelet count, neutrophil count, lymphocyte count, neutrophil to lymphocyte ratio, platelet to lymphocyte ratio, blood pH, bicarbonate sodium level (HCO3), serum sodium, serum potassium, serum calcium, albumin, weight (kg), BMI (kg/m2), body fat percentage, calculated via Dual-energy X-ray absorptiometry (DXA). Echocardiography with epicardial adipose tissue (EAT) assessment, bilateral carotid intima media thickness (IMT), carotid resistive index via carotid Doppler, flow mediated dilation (FMD) and renal resistive index (RRI), were recorded. The postoperative values were all recorded 1 year after bariatric surgery.

The study was approved by the Ethical Committee of this University hospital. All participants provided written informed consent to participate in the study. Additional written informed consent was obtained before all surgical procedures. All methods were carried out in accordance with relevant guidelines and regulations.

### Postoperative follow-up

After surgery, an upper gastrointestinal series with a water-soluble iodinated contrast was routinely performed 2 days postoperatively. Patients were discharged on a semiliquid diet for 1 month along with 30 mg daily of PPIs for the first 3 months, followed by 15 mg for the next 2 months and discontinued if the patient was asymptomatic for GERD. Follow-up schedule entailed medical examination at 1, 3, 6 and 12 months after surgery and once a year thereafter.

Follow-up was fully completed 1 year after surgery by all patients.

### Sleeve gastrectomy surgical technique

Our standard surgical procedure for SG has been previously described [13]. All surgical procedures were performed laparoscopically by a 5-trocar technique. Full mobilisation of the gastric fundus with complete dissection of the posterior gastric wall from the left diaphragmatic crus was achieved. A 48-Fr calibration orogastric bougie is routinely used. Resection was begun approximately 6 cm from the pylorus and continued cephalad reaching the angle of His. A gastric sleeve with a residual capacity of 60–80 mL (as measured by administration of methylene blue saline solution via a nasogastric tube) was achieved.

### Echocardiography and epicardial adipose tissue assessment

All patients underwent transthoracic echocardiography with a commercially available cardiovascular ultrasound system (Vivid E9, GE, Horten, Norway). Measurements of cardiac chambers were made according to established criteria. Left ventricular ejection fraction, by modified biplane Simpson method, and left ventricular mass index (LVMI) were estimated. Peak early (E) and late (A) diastolic velocities, deceleration time, left ventricular isovolumic relaxation time, and myocardial performance index were obtained using standard Doppler practices. Standard parasternal, apical, and subcostal views were used. We performed the measurement of the EAT perpendicularly on the free wall of the right ventricle at the end of the diastole in the parasternal long-axis view in three cardiac cycles.

### Renal resistive index

Participants were studied with the high-resolution B-mode ultrasound machine Toshiba Aplio XV (Toshiba AplioxV, Toshiba American Medical Systems, Inc., Tustin, CA, USA) equipped with a 3–3.5 MHz convex transducer. All measurements were made by a single, blinded, experienced ultrasonographer. We used an anterior approach, in the prone position, and an oblique approach, in lateral position, for detecting the renal arteries and intra-parenchymal vessels. The interlobular, interlobar or arcuate arteries in both kidneys were identified by colour-flow imaging and blood-flow profile in the artery was monitored by spectral analysis. RRI values were determined with the mean of three separate measurements in the renal superior pole, interpolar regional and inferior pole in both kidneys. These measurements were used to calculate the average RRI value for each kidney, and then the average RRI value for each patient was calculated as the mean of the RRI in the left and right kidney. We determined the peak systolic velocity and end-diastolic velocity (centimetres/seconds) to calculate the renal resistive index (RRI) as = [1-(end-diastolic velocity ÷ maximal systolic velocity)] × 100.

### Flow-mediated dilation brachial artery

According to the method described by Corretti et al. [14], the endothelium-dependent vasodilation (flow-mediated dilation [FMD]) of the brachial artery was assessed using a high-resolution B-mode ultrasound machine Toshiba Aplio xV equipped with a 5- to 12 MHz linear transducer with a 0.01-mm resolution, by the same blinded experienced ultrasonographer, following a standardized protocol [15]. This measurement was not assessable in HD patients. FMD was expressed as the change in post stimulus diameter as a percentage of the baseline diameter. FMD: (diameter post hyperaemia basal diameter/basal diameter) £ 100. The values of FMD were considered normal if >10 %.

### Common carotid intima-media thickness assessment

Right and left carotid ultrasound was blindly performed by an experienced sonographer (S.L.) who was unaware of the characteristics of the patients under examination. Participants were studied with the high-resolution B-mode ultrasound machine, Toshiba Aplio XV (Toshiba Aplio XV, Toshiba American Medical Systems, Inc., Tustin, CA, USA) equipped with a 5- to 12-MHz linear transducer with a 0.01-mm resolution, following a standardized vascular protocol. IMT was measured at three points on the far walls of both left and right distal common carotid arteries, carotid bulb, and the proximal portion of the internal carotid arteries [16]. The mean IMT was computed as the average IMT on both sides. The value of IMT was considered normal when between 0.55 and 0.9 mm.

### Statistical analysis

Variables are presented as mean ± standard deviation and compared using t-paired test. Statistical analysis was performed using IBM® SPSS® Statistics version 27. The confidence interval was set to 95 %, and p was considered significant at <0.05.

## Results

A total sample size of 24 patients was analysed, 12 males, mean age 38.1 ± 2.5 years. Mean BMI pre-operatively was 41.8 ± 4.2 kg/m^2^. In Table 1 pre-operative values and post-operative values are compared. Results were summarised into categories in relation to cardiovascular, renal and metabolic parameters.

**Table 1:** Mean value, standard deviation, *t-*test and *p* value.

	Baseline	1 year follow up	*t-*test	p value
Creatinine mg/dL	0.79 ± 0.14	0.8 ± 0.19	−0,368	0,716
eGFR ml/min	102,03 ± 16.57	99,83 ± 19.37	0,722	0,478
Uricemia mg/dl	5,57 ± 2.39	5,39 ± 2.1	0,244	0,810
Urea mg/dl	35,29 ± 9	35,44 ± 11.42	−0,057	0,955
Glycemia mg/dl	100,32 ± 32.12	86,04 ± 12.8	2926	0,008
Insulinemia μU/ml	19,01 ± 8.22	12,55 ± 5.66	4698	<0,001
HOMA-IR	4,81 ± 2.67	2,72 ± 1.35	4439	<0,001
Total Cholesterol mg/dL	183,74 ± 33.21	172,27 ± 20,65	1516	0,145
LDL-C mg/dl	110,11 ± 29.08	98,6 ± 19.51	1862	0,077
HDL-C mg/dl	45,17 ± 9.4	51,24 ± 8.42	−3501	0,002
TG mg/dl	140,93 ± 69.9	108,40 ± 39.7	1956	0,065
PTH pg/dl	59,95 ± 24.2	58,8 ± 21.43	−0,262	0,797
Vitamin D3 ng/dl	15,44 ± 7.39	29,28 ± 10.07	−5362	<0,001
CRP μg/dl	20,210,1 ± 34,359,53	2769,00 ± 3039,04	2381	0,028
Haemoglobin g/dl	14,12 ± 1.12	13,88 ± 1031	0,932	0,361
MCV fL	87,81 ± 4.33	89,46 ± 3,16	−1484	0,158
MCHC g/dl	32,71 ± 0.99	32,71 ± 0.99	−0,206	0,839
Hct %	43,15 ± 3.34	43 ± 2.88	0,563	0,579
RBC μl	4,934,782,61 ± 325,169,46	4,674,608,7 ± 385,348,45	58,217	<0,001
GB μl	7001,70 ± 2540.27	6986,91 ± 1919,02	0,033	0,974
PLT μl	239,043,48 ± 75,163.75	208,321,74 ± 67,072,14	1913	0,069
Neutrophil count μl	4801,36 ± 1645.07	3848,64 ± 1285,95	3158	0,005
Lymphocite count μl	2229,55 ± 611.98	2590 ± 616,25	−2406	0,025
Neutrophil/lymphocite ratio	2,19 ± 0.65	1,54 ± 0,69	3365	0,003
Platelet/lymphocit ratio	113,08 ± 44.09	84,97 ± 37,34	2230	0,037
pH	7,42 ± 0.03	7,41 ±,027	1101	0,284
Serum bicarbonate mmol/l	25,896 ± 2.08	27,47 ± 2,27	−2023	0,05
Serum sodium mmol/l	141,96 ± 3.4	141,04 ± 26,681	1115	0,277
Serum potassium mmol/l	4,3 ± 0.35	4,25 ± 0,42	0,932	0,362
Serum calcium mmol/l	2,4 ± 0.11	2,12 ± 0,45	3048	0,006
Albuminaemia g/l	41,9 ± 8.5	42,02 ± 8,4	−0,188	0,852
Transferrine g/l	2,54 ± 0.29	2,54 ± 0,33	−0,053	0,959
Weight kg	119,84 ± 21.92	85,68 ± 12,84	10,252	<0,001
BMI kg/m^**2**^	41,64 ± 4.46	30,48 ± 4,7	11,394	<0,001
Hand grip kg	29,47 ± 9.13	31,13 ± 9,42	−1477	0,153
EAT	10,33 ± 3.13	6,8 ± 1,48	7415	<0,001
Fat mass %	41,03 ± 6.62	31,05 ± 7,2	11,607	<0,001
Right IMT	0,83 ± 0.17	0,76 ± 0,18	1728	0,096
Left IMT	0,84 ± 0.17	0,71 ± 0,22	2807	0,009
Right RRI	0,65 ± 0.04	0,61 ± 0,04	3319	0,003
Left RRI	0,64 ± 0.05	0,62 ± 0,05	2202	0,037
FMD	11,25 ± 6	16 ± 7,03	−3646	0,001

BMI: body mass index; CRP: C-reactive protein; eGFR: estimated glomerular filtration rate; EAT: epicardial adipose tissue; FMD: flow-mediated dilation; HDL: high density lipoprotein; HOMA-IR: Homeostatic Model Assessment for Insulin Resistance; IMT: intima media thickness; LDL: low density lipoprotein; MCHC: Mean Corpuscular Haemoglobin Concentration; MCV: Mean Corpuscular Volume PTH: parathyroid hormone; RBC: red blood cells; RRI: renal resistive index; TG: thyroglobulin.

### Cardiovascular outcomes

Nine measures of cardiovascular effects were recorded within the cohort both at the preoperative stage and 1-year post-operatively. These measures included laboratory values as well as physical cardiac and endothelial evaluations. Lipid profile showed that the only component with significant improvement was HDL cholesterol (*p* = 0.002).

While total cholesterol, LDL cholesterol and triglycerides still showed improvement, none of the findings were significant (Table 1). EAT was recorded preoperatively and postoperatively. Our analysis showed a significant improvement at 1 year follow up (*p* < 0.001). The IMT of both the right and left carotid arteries in each of our patients was also recorded and analysed and showed a significant improvement 1 year after surgery (*p* = 0.003) on the left. Lastly, FMD values showed an improvement at the 12-month postoperative control (*p* = 0.001).

### Renal outcomes

The renal outcomes of bariatric surgery at one year were measured via comparison of creatinine levels, eGFR, urea and uricemia levels. None of the renal outcomes had statistically significant improvements after bariatric surgery.

Left and right RRI showed a statistically significant decrease compared to preoperative levels (*p* = 0.037 and *p* = 0.003). Secondary renal outcomes including Vitamin D3 (*p* < 0.0001), calcium levels (*p* = 0.006), bicarbonate levels (*p* = 0.05) and RBCs (*p* = 0.007) all showed significant improvements postoperatively.

### Inflammatory markers

Inflammatory markers that showed statistically significant improvements 1 year after SG include: RCP (*p* = 0.028), neutrophil count (*p* = 0.005), neutrophil:lymphocyte ratio (*p* = 0.003), lymphocyte count (*p* = 0.025) and platelet:lymphocyte ratio (*p* = 0.037).

### Glycaemic control

With regards to glycaemic control at 1-year postoperative follow-up, blood glucose levels (mg/dl) within our patient cohort had a statistically significant improvement (*p* = 0.008). Insulin levels also improved significantly at 1-year follow-up post bariatric surgery (*p* < 0.001). Values for HOMA-IR were also compared, showing significant improvement in hepatic insulin resistance at 1 year after surgery (*p* < 0.001).

### Weight loss/strength outcomes

As expected, BMI had marked improvement after SG (p < 0.001), along with total weight (p < 0.001) and body fat percentage (p < 0.001). An additional marker of health in the form of hand grip strength was added, which rather than showing an improvement showed a decline but was not statistically significant (*p* = 0.153). However, there was a significant positive correlation between postoperative hand grip and both insulinemia and HOMA-IR at 1-year follow-up.

## Discussion

The present study aimed at analysing the effect of BS, and more in particular of SG, on cardiovascular, renal and metabolic parameters in obese patients.

We found substantial improvement in several cardiovascular parameters, including HDL-cholesterol, IMT, EAT and FMD. Of note, it is significant the reduction of IMT and FMD which reflects the early beneficial effects of SG on endothelial function.

A major role in cardiovascular disease is also played by glucose homeostasis which is well-known to significantly improve after BS [17]. Levels of blood glucose, insulin and HOMA-IR confirmed the effectiveness of SG also from this point of view, improving insulin resistance, reducing the consequential risk of endothelial dysfunction, atherogenic lipid profile, and new hypertension onset [18].

With regards to renal outcomes, a significant increase in vitamin D3, Calcium, RBCs and concurrent reduction of bicarbonate levels was recorded. Additionally, RRI significantly improved 1 year after surgery. To detect vessel impairment early and effectively, as well as to determine the best course of therapy, it is crucial to analyse macro and microvascular circulation. This analysis is also very important for the primary and secondary prevention of cardiovascular illnesses. By using Doppler sonography to calculate the resistive index, impedance, and renal vascular resistance and, ultimately, arteriolar damage could be evaluated. Furthermore, increased RRI, a marker of augmented microvascular tone, correlates with the degree of kidney damage determined by high blood pressure, also known as hypertensive nephrosclerosis, in the context of cardiovascular risk. RRI has been linked to several cardiovascular risk factors, including carotid atheromatosis and left ventricular hypertrophy, in numerous investigations [[19], [20], [21]]. Significant connections between renal resistance and aortic stiffness, as determined by both pulse wave velocity and central pulse pressure, have also been observed [22]. Despite our cohort of patients not yet displaying parameters of renal damage, given the young age, we found a significant improvement in renal microvascular tone as reflected by RRI. The early execution of bariatric surgical procedures might indeed contribute to preventing renal injury in those subjects affected by morbid obesity.

Inflammatory indexes were also analysed and showed an overall significant reduction.

Specifically, CRP, neutrophils, lymphocytes, neutrophil:lymphocyte and platelet:lymphocyte ratios presented a substantial decrease compared to preoperative values. A systemic low-grade chronic inflammatory condition that is characterised by a rise in systemic inflammatory markers frequently coexists with obesity [23]. It is known in fact the unique pro-inflammatory environment sustained in this population, with an increased activation of innate and adaptive immune responses [24], contributing to the development of major cardiovascular risk factors, such as hypertension, dyslipidemia and endothelial dysfunction [25].

Various chronic inflammatory cytokines and a nonspecific immune system activation are thought to play a significant role in the development of obesity-related diseases. The low-grade systemic inflammation induced by obesity has been shown to be concurrently responsible for the development of T2DM, non-alcoholic fatty liver disease, asthma, various types of cancer, cardiovascular and neurodegenerative diseases [26,27]. The substantial reduction of several inflammatory markers that we have shown has then deep interrelation to the improvement of the metabolic parameters assessed above.

In conclusion, we have shown the positive impact of BS, translating into reduction of adipose tissue mass and systemic inflammation and endothelial dysfunction, all leading to improvement in insulin resistance, cardiovascular disease and progressive kidney damage, to the point where premorbid conditions as diabetes and chronic renal disease may undergo partial or complete remission postoperatively, when treated pre-emptively.

## Funding

This research received no external funding.

## Ethics approval and consent to participate

the methods were carried out in accordance with relevant guidelines and regulations. The experimental protocol was ap0proved by the Ethical Committee of the Department of Surgical Sciences at Sapienza University. Informed consent was obtained from all participants.

## CRediT authorship contribution statement

**Maria Irene Bellini:** Conceptualization, Formal analysis, Methodology, Writing – original draft. **Lidia Castagneto Gissey:** Conceptualization, Data curation, Investigation, Methodology, Writing – review & editing. **Denise V. Nemeth:** Visualization, Writing – original draft. **Vito D'Andrea:** Supervision, Writing – review & editing. **Giulio Illuminati:** Supervision, Validation, Writing – review & editing. **Serena Marchitelli:** Investigation, Resources, Writing – review & editing. **Silvia Lai:** Data curation, Methodology, Writing – review & editing. **Giovanni Casella:** Data curation, Investigation, Methodology, Writing – review & editing.

## Declaration of competing interest

The authors declare no conflict of interest.
